# Effect of the iridocorneal angle size on the diurnal pressure profile in a glaucoma suspect cohort and patients with glaucoma

**DOI:** 10.1007/s10792-023-02823-x

**Published:** 2023-09-02

**Authors:** Michael Müller, Ana Pavlovic, Theresa Niermann, Ivana Pavlovic, Siegfried Priglinger, Thomas Kohnen, Mehdi Shajari, Marc Mackert

**Affiliations:** 1 Department of Ophthalmology, Goethe-University, Theodor-Stern-Kai 7, 60590 Frankfurt am Main, Germany; 2 Department of Ophthalmology, Ludwig-Maximilians University, Munich, Germany

**Keywords:** Iridocorneal angle, Glaucoma, Intraocular pressure (IOP), Visual field defects, Pentacam (Oculus)

## Abstract

**Purpose:**

To evaluate the impact of the iridocorneal angle size (ICAS) on the diurnal intraocular pressure (IOP) in patients with suspected glaucoma (SG).

**Method:**

Patients with any eye-pressure lowering medication or previous ocular surgery were excluded. In a retrospective study set, diurnal IOP profiles of 120 patients (205 eyes) within a 48-h period were analysed by regression analysis. Of those eyes, 44 were diagnosed to have glaucoma. The remaining eyes were used as healthy control group (HCG).

**Results:**

The overall mean IOP was 15.63 mmHg ± 2.72 mmHg and mean ICAS was 23.92° ± 4.74°. In the glaucoma cohort, mean IOP was 18.77 ± 1.86 mmHg and mean ICAS was 25.02° ± 4.96°. In the HCG, mean IOP was 14.77 ± 2.25 mmHg and mean ICAS was 23.62° ± 4.64°. In the total cohort, as well as in the subgroups (HCG or glaucoma), regression analysis showed no significant impact even of the minimum ICAS, which was larger than 10°, on average (*P* = 0.89), maximum (*P* = 0.88), and range of IOP (*P* = 0.49) within 48 h. The difference between glaucoma cohort and HCG cohort was significant in terms of IOP (*P* < 0.001), but not for minimum ICAS (*P* = 0.07). Chi-square test showed no increase in prevalence of IOP peaks of  > 21 mmHg within 48 h in eyes with an angle between 10° and 20° (*P* = 0.18).

**Conclusion:**

An ICAS of larger than 10° in HCG or glaucoma patients with an open-angle does not influence the minimum, average, maximum or range of IOP. Additionally, an angle size larger than 10° does not allow the prediction of IOP changes in these two cohorts.

## Introduction

In the past, primary glaucoma was simply defined as “an elevated IOP over the normal range (21 mmHg)” and the appearance of an increased excavation of the optic nerve head and visual field defects [1–4]. The modern, more accurate definition includes the physiological changes caused by glaucoma: it’s a “chronic neuropathy leading to specific changes in the morphology of the optic disc, the reduction of retinal nerve fibres, and the loss of ganglian cell function form visual field defects” [5–8]. From a more anatomical perspective, primary glaucoma is typically grouped into two main categories due to the different characteristics of the cornea and iris-basis formation, the so called iridocorneal angle (ICA): primary open-angle (POAG) and primary angle-closure glaucoma (PACG) [9], where the latter seems to be more often connected with IOP peaks.

Due to the variety of “normal” changes in the optic disc morphology and the variability in measurements of the RNFL, it is challenging in suspected cases to detect early functional defects [6, 7] and to decide, if the eye shows just a variation of the norm or is already in an early stage of glaucoma.

Although the definition of glaucoma has changed, an elevated IOP is still one of the most important risk factors and helps to diagnose potential glaucoma. In order to detect an elevated IOP in patients with suspected glaucoma, it is often not enough to measure the IOP once, because it is well known that the IOP is changing over time. Thus, repeating measurements over the day, a so-called diurnal tension curve (DTC), is an appropriate method to detect possible IOP peaks and to help to decide if an early stage of glaucoma is present. Thus, in patients with SG and so far normal IOP we are used to add a DTC to our diagnostic armamentarium. Due to the fact that it is difficult to include a night-time measurement to a DTC in an out-patient setting we perform DTCs during an in-patient session.

In an effort to combine all relevant data points to detect a glaucoma, our out-patient department performed measurents of the IOP in a sitting position with applanating technique (Goldmann), a corneal thickness measurement (Pentacam, Oculus, Wetzlar, Germany) to correct the IOP, a visual field test (program 32/2, Octopus 900, Haag Streit, Switzerland), a gonioscopy, a photo and an OCT-measurement (Topcon OCT 2000, Japan) of the optic disc. The clinical diagnostics is complemented by a 48-h pressure profile (DTC) on an in-patient situation.

Although DTCs are relevant to detect early stages of glaucoma, it is worth noting that reportings of diurnal IOP changes are inconsistent and unpredictable: some studies report a morning IOP peak [10–13], whereas other studies show higher nocturnal IOP levels [14, 15]. Furthermore, several parameters seem to have an influence on the diurnal pressure profile: physical activity, fluid intake, chronobiological rhythms [16] and interestingly, the time of the year [17]. Despite all these influencing factors, the 48-h measurement enables us to evaluate the baseline IOP-level, diurnal IOP changes and IOP peaks and lightens the decision between a healthy eye situation or a glaucoma.

Concerning the diagnostics of glaucoma and due to the differentiation of POAG and PACG on the basis of clinically ICA evaluation, we were interested if different anterior chamber angles (ACA) would have an effect on diurnal pressure profiles in patients with suspected glaucoma (SG). Our hypothesis was that a smaller anterior chamber angle could result in a higher average IOP and a higher frequency of pressure peaks (IOP > 21mmHG).

This retrospective study evaluates the influence of the iridocorneal angle size (ICAS), measured by a rotating Scheimpflug camera (Pentacam, Oculus, Wetzlar, Germany), on the IOP during a diurnal intraocular pressure profile in SG eyes without any IOP lowering therapy. In a further subgroup analysis, patients in our cohort who were diagnosed to have glaucoma due to their IOP profile in combination with other existing data were considered as a glaucoma-group and compared to the remaining “healthy” cohort.

## Materials and methods

In this retrospective study, 120 SG in-patients (205 eyes) were analysed. All patients were recruited between 2014 and 2018 at the Clinic of Ophthalmology, Goethe University, located in Frankfurt am Main, Germany. The study was approved by the local ethics committee and is in accordance with the Helsinki Declaration.

Exclusion criteria were any prior medical or surgical intervention for the control of IOP, past ocular surgery, any other intraocular disorder, condition preventing reliable applanation tonometry or visual field assessment. None of the patients were on any systemic or topical medication that could have potentially influenced the IOP. Patients were specifically asked about former use of topical or systemic steroids.

To evaluate the effects of the iridocorneal angle size (ICAS) on the IOP, patients underwent diurnal intraocular pressure measurements (DTC). Within a 48-h in-patient period, a total of 10 measurements per patient were performed. Every day 5 measurements were performed at 8 a.m., 12 p.m., 4 p.m., 8 p.m. and 10 p.m.. All measurements were performed in sitting position without using pupil size changing eyedrops and under photopic light conditions. IOP was measured using the Goldmann applanation tonometry (GAT) by the respective ophthalmologist, who has been trained in such a procedure. The tonometer was attached to a slit-lamp. Because IOP measurements by GAT are altered by the central corneal thickness (CCT), we used the Dresdner correction table to achieve an “IOP corrected for corneal thickness” [18, 19].

The final decisions whether the patient had glaucoma or not were made by our senior ophthalmologist. The decision-making criteria were clinical appearance of optic nerve head, appearance of iridocorneal angle in gonioscopy, the IOP profile, visual field examination (Octopus 900), and the RNFL measured by OCT (Topcon 3D OCT-2000) in accordance with the “Terminology and Guidelines for Glaucoma” of the European Glaucoma Society (www.eugs.org) [20]. Here, a papilla excavation of more than 0.5 in combination with beginning visual field defects or a reduced RNFL (retinal nerve fibre layer) and an IOP elevated over 21 mmHg in the DTC lead to the diagnosis of glaucoma. The final diagnosis, including whether therapy would be necessary or not, was made before the patients were discharged.

We obtained measurements of the iridocorneal angle (ICA) and anterior chamber depth in a darkened room using a rotating Scheimpflug camera (Pentacam, Oculus, Wetzlar, Germany) in sitting position without using pupil size changing eyedrops. Pupil size was measured by the device. The ICAS was measured manually in the superionasal, nasal, inferior nasal, inferior, inferiotemporal, temporal, and superiotemporal angle. The technique and advantage of the manual measurement of the ICA was described in detail by Shajari et al. in 2019 [21]. The superior ICA was not included in our evaluation because it is often masked by the upper lid and thus the data is incorrect. A screenshot of the Pentacam measurement was obtained, printed, and then evaluated using a square and angle meter. The technician ensured that the measuring line was drawn tangentially from the trabecular meshwork/scleral spur to the anterior iris surface, and tangentially to the inner corneal surface. The received ICAS was measured using an angle meter (Table 1). All measurements of the ICAS were completed by the same person.

**Table 1:** Specifications of the healthy cohort and eyes with glaucoma

Variable	HCG (*n* = 161)				Glaucoma (*n* = 44)				*P* = *
	Mean	Std. Dev	Min	Max	Mean	Std. Dev	Min	Max	
Age (years)	49.56	17.73	15	86	50.77	16.76	15	76	0.5907
Min IOP (mmHg)	12.27	2.08	7	17	15.53	2.29	11	21	** < 0.0001**
Average IOP (mmHg)	14.77	2.25	8	20	18.77	1.86	14	25	** < 0.0001**
Max IOP (mmHg)	17.71	2.48	11	21	23.30	1.83	21	28	** < 0.0001**
IOP Range (mmHg)	5.44	1.96	2	11	7.75	2.38	3	14	** < 0.0001**
MD 30 (dB)	2.40	3.43	-2.1	13.4	3.51	3.35	-4.3	12.6	**0.0017**
sLV 30 (dB)	3.04	1.86	1.2	8.7	3.50	2.10	0.2	10.3	**0.0293**
RNFL	96.93	14.22	41	133	93.34	15.11	57	116	0.1646
Rim volume (mm^3^)	0.36	0.25	0.01	1.83	0.26	0.23	0	1.41	**0.0026**
IC Angle Min (°)	21.54	4.80	11	34	23.23	5.04	12	33	0.0755
IC Angle Average (°)	23.62	4.64	14	35	25.02	4.96	13	34	0.1389
IC Angle Max (°)	25.81	5.06	15	40	26.70	5.20	15	38	0.3942
Anterior chamber depth (mm)	2.81	0.41	1.88	4.02	2.97	0.48	1.81	4.24	0.0541
Anterior chamber volume (mm^3^)	160.01	39.82	79.4	315.5	164.72	39.70	69.4	263.3	0.5225
Corneal thickness (µm)	550	33.16	466	652	531	40.59	389	608	**0.0062**
Pupil (mm)	3.55	1.4	1.57	7.52	3.01	0.80	1.56	6.50	0.0785

*Min IOP* lowest IOP measured within 48 h, *IOP Range* difference between the lowest and highest IOP measured within 48 h, *IC Angle Min* smallest iridocorneal angle measured in one eye

*Statistical mean comparison (Welch *t* test or Wilcoxon–Mann–Whitney test depending on data distribution); bold numbers are statistically significant (*P* < 0.05)

For statistical analysis, the Shapiro–Wilk or Kolmogorov–Smirnov test was used to analyse data distribution. Depending on this outcome, a parametric *t* test or a non-parametric Wilcoxon–Mann–Whitney test was used to compare means between the glaucoma and the healthy eye subgroups. Under the assumption that ICAS influences the IOP, the smaller the ICAS the higher this effect would be. Thus, linear regression analysis was performed to detect the impact of ICAS on minimum, average, maximum, and range (defined as difference of maximum and minimum pressure during the 48 h pressure profile) of IOP. *P* < 0.05 was considered statistically significant. Because including both eyes of the same patient can be viewed as a source of bias, we additionally analysed only the right eye of each patient to evaluate inter-collinearity. Further, in a subgroup analysis we compared the obtained glaucoma-group to the remaining “healthy” cohort.

## Results

Diurnal intraocular pressure profiles of 120 SG patients (total of 205 eyes), without any IOP lowering therapy, were analysed.

For all eyes, the overall mean IOP was 15.63 mmHg ± 2.72 mmHg (range 8–25 mmHg) and the mean iridocorneal angle size was 23.92° ± 4.74° (range 13°–35°). We did not register an iridotrabecular contact (ITC) with two or more quadrants during the clinical assessment.

**Fig. 1:**
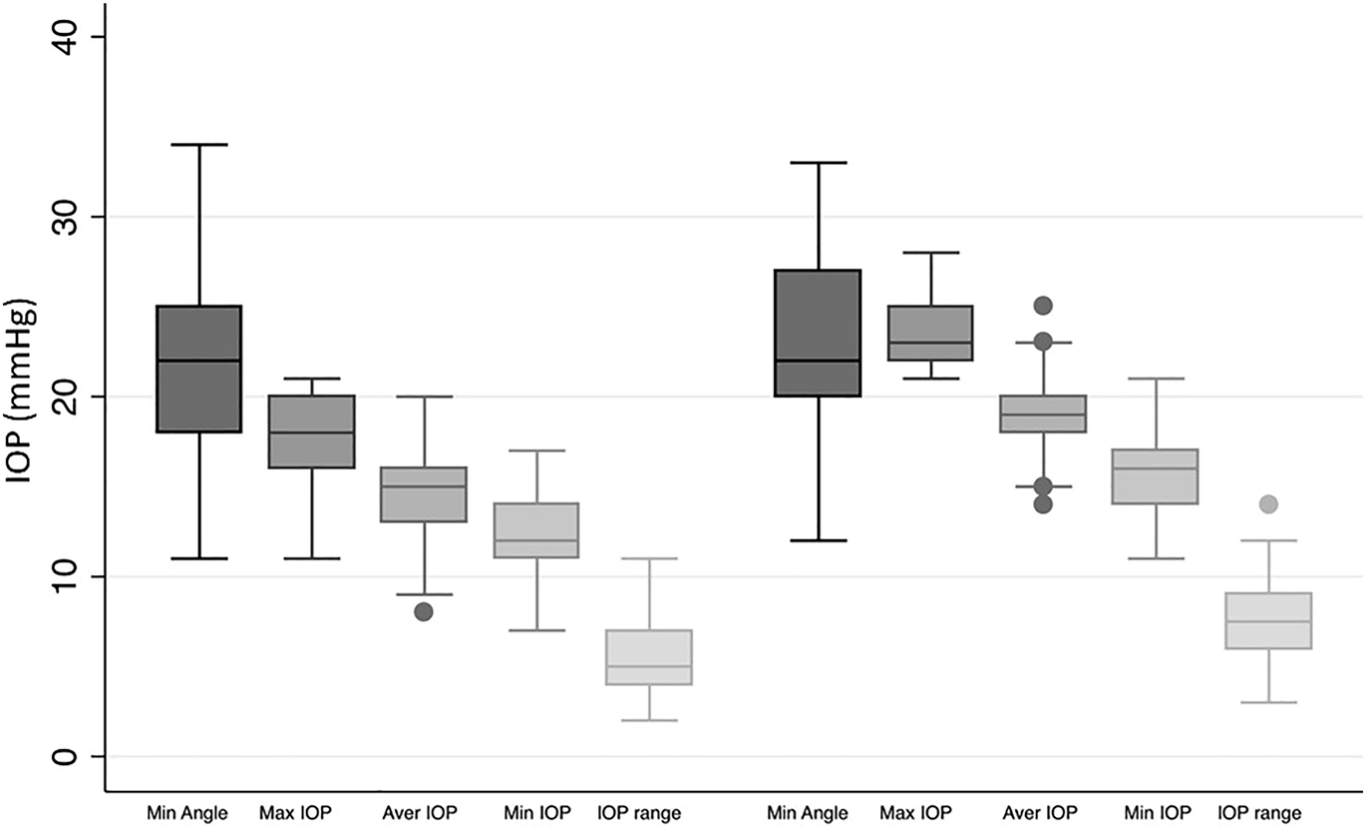
Boxplot of the minimum iridocorneal angle as well as minimum, average, maximum IOP and IOP range in a HCG (on the left) and eyes with glaucoma (on the right). It is visible that there is no significant difference in terms of the iridocorneal angle, but a noticeable difference in IOP

A regression analysis was performed to detect the effect of the angle size on the IOP: In the total examined population, it showed no significant impact even of the minimum ICAS, which was larger than 10°, on average IOP (*P* = 0.89), maximum IOP (*P* = 0.88), and range of IOP (*P* = 0.49) within 48 h (Fig. 1).

**Fig. 2:**
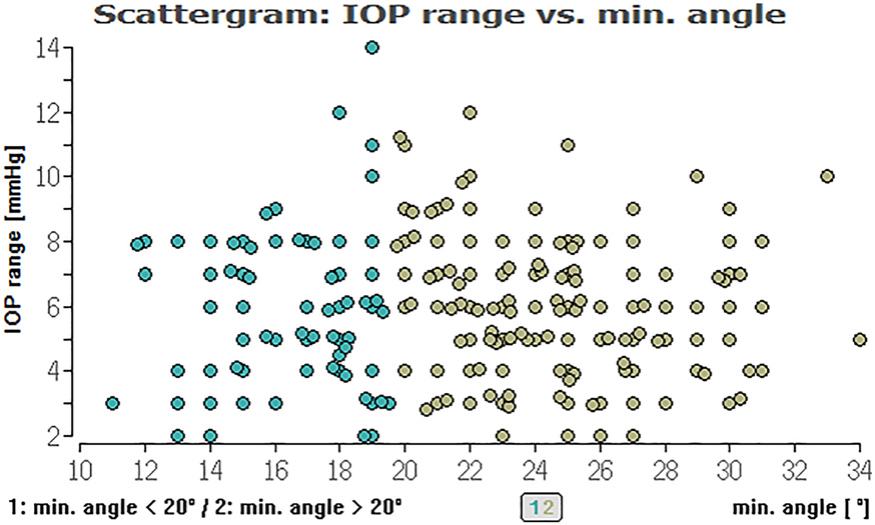
Distribution of IOP range and minimum ICAS for all eyes regarding an ICAS < 20° (green) and ICAS > 20° (khaki). No significant impact of the minimum iridocorneal angle on IOP for all eyes

For the overall cohort, regressing analysis showed for the minimum (*P* = 0.73), the maximum (*P* = 0.95), average ICAS (*P* = 0.79) as well as for the angle variance (*P* = 0.53) no significant impact on the maximum IOP. This also applies to the mean IOP: The minimum (*P* = 0.81), the maximum (*P* = 0.90), and the average ICAS (*P* = 0.90), as well the angle variance (*P* = 0.42), showed no significant impact on the mean IOP (Fig. 2).

We additionally performed the regression analysis only for the right eye of each patient to evaluate the inter-collinearity. It showed that for the whole cohort even the minimum ICAS has no impact on average (*P* = 0.44), maximum (*P* = 0.69) and range of IOP (*P* = 0.77).

**Fig. 3:**
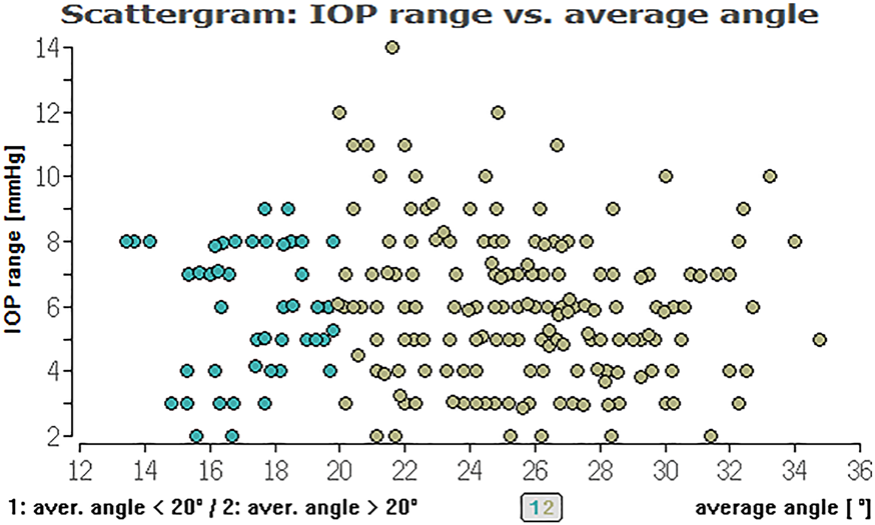
Distribution of IOP range and average ICAS for all eyes regarding an ICAS < 20° (green) and ICAS > 20° (khaki). No significant impact of the average iridocorneal angle on IOP for all eyes

**Fig. 4:**
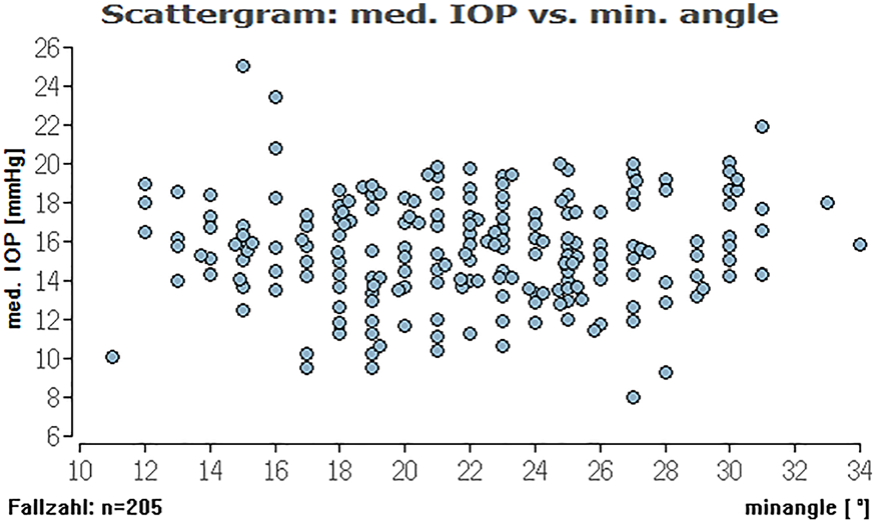
Distribution of medium IOP and minimum ICAS for all eyes. No significant impact of the minimum iridocorneal angle on medium IOP for all eyes

**Fig. 5:**
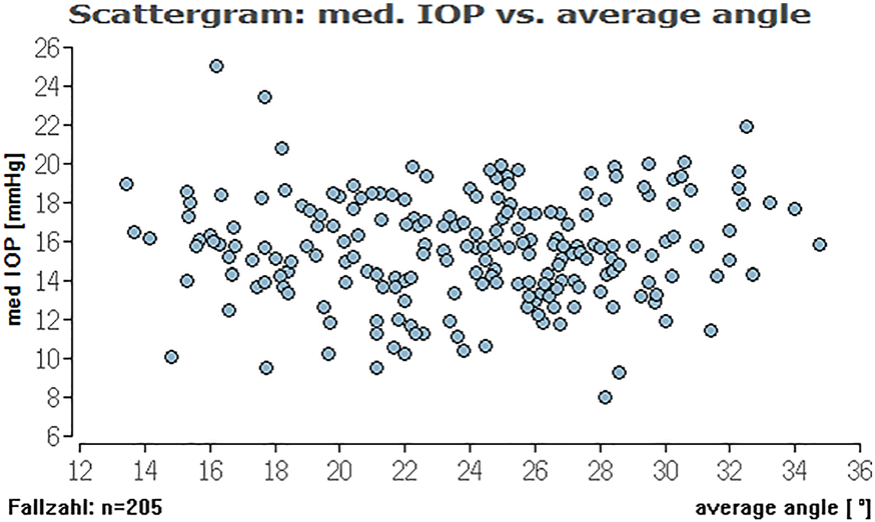
Distribution of medium IOP and average ICAS for all eyes. No significant impact of the average iridocorneal angle on medium IOP for all eyes

**Fig. 6:**
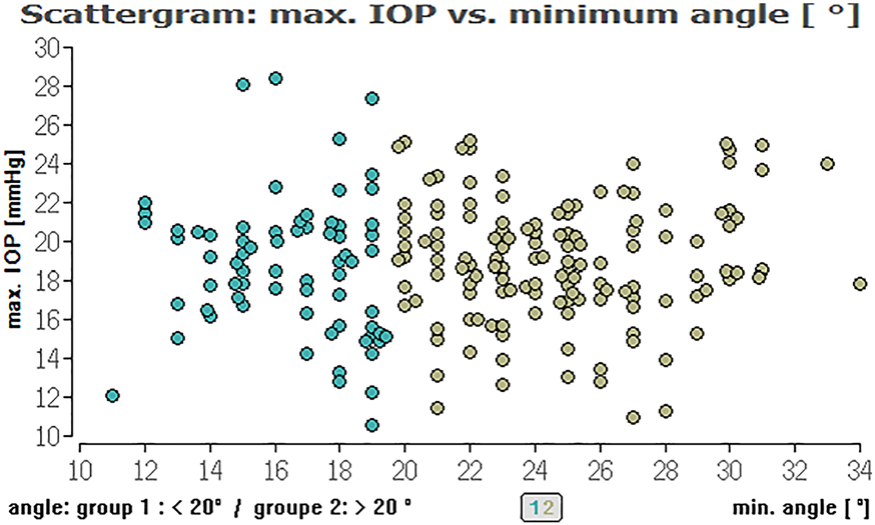
Distribution of maximum IOP and minimum ICAS for all eyes regarding an ICAS < 20° (green) or ICAS > 20° (khaki). No significant impact of the minimum iridocorneal angle on maximum IOP for all eyes

**Fig. 7:**
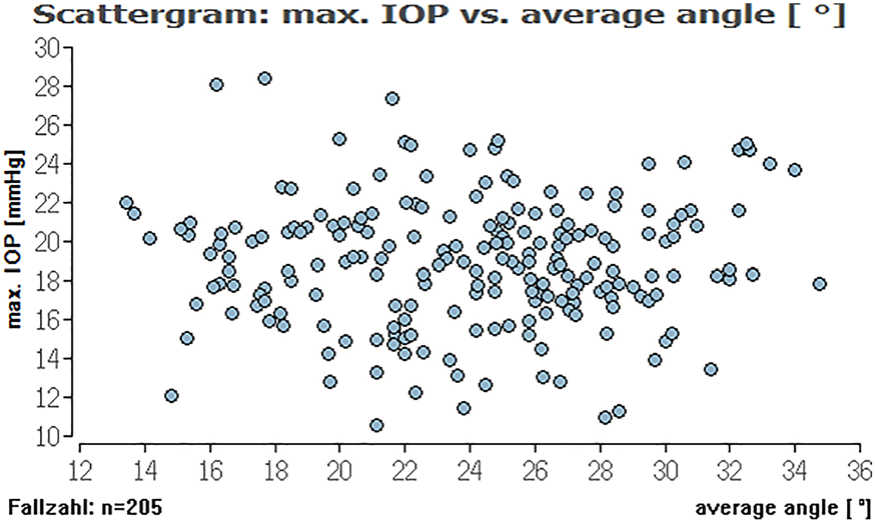
Distribution of maximum IOP range and average ICAS for all eyes. No significant impact of the average iridocorneal angle on maximum IOP for all eyes

**Fig. 8:**
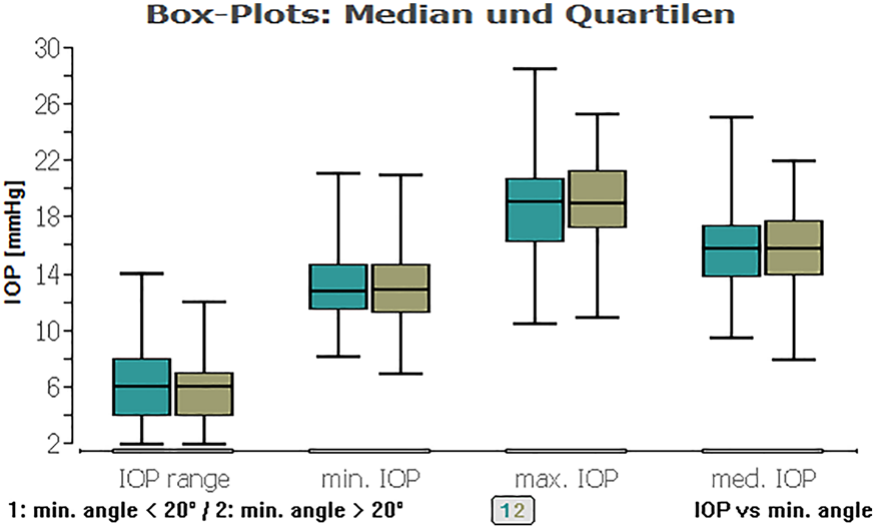
Boxplot of IOP types (range, min., max., and med.) and a minimum ICAS < 20° (green) or ICAS > 20° (khaki). No significant impact of minimum ICAS smaller or larger 20° within IOP types for all eyes

**Fig. 9:**
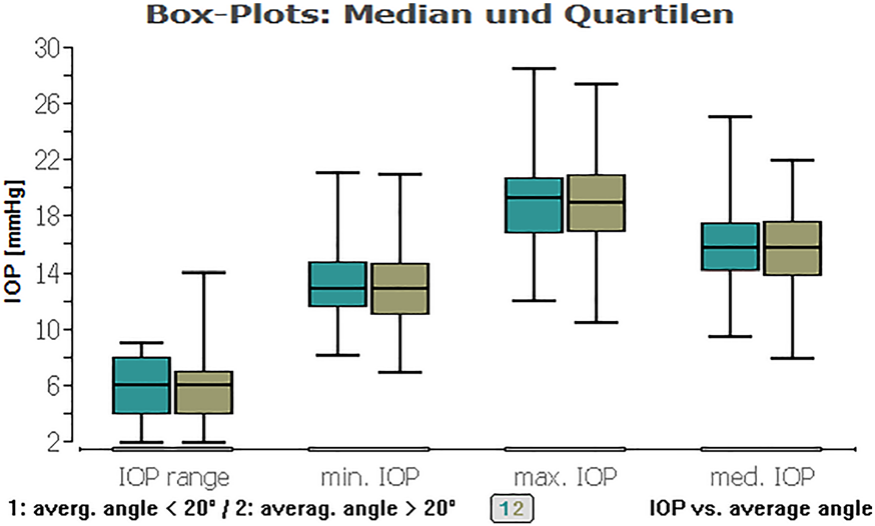
Boxplot of IOP types (range, min., max., and med.) and an average ICAS < 20° (green) or ICAS > 20° (khaki). No significant impact of average ICAS smaller or larger 20° within IOP types for all eyes

To evaluate, if there is an (linear) effect of ICAS in the range of 10° to 20° and an ICAS of larger than 20° on different types of IOP we calculated Pearson correlations between these parameters (Table 2). Here, we could not find a relevant linear correlation between the different IOPs (range, minimum, median, maximum) and ICAS (minimum or average angle) (see Figs. 2, 3, 4, 5, 6, 7; boxplots Figs. 8, 9).

**Table 2:** Pearson correlation between IOPs (range, min., med. and max.) and ICAS (min. and average) regarding all angles or angles smaller or larger 20°

IOP	ICAS	Angle (°)	Numbers *n* =	Correlation coefficient *r* =	Exceeding probability *P* =	CI (*P* = 0.95)	CI (*P* = 0.95)
Range	Minimum	All	205	− 0.0352	0.616745	− 0.1714	0.1024
Min				0.0707	0.313519	− 0.0669	0.2058
Med				0.0336	0.63.281	− 0.1039	0.1698
Max				0.0298	0.671809	− 0.1077	0.1661
Range	Average	All	205	− 0.068	0.332873	− 0.2031	0.0697
Min				0.0862	0.218965	− 0.0514	0.2207
Med				0.0231	0.742312	− 0.1143	0.1596
Max				0.019	0.786807	− 0.1183	0.1556
Range	Minimum	< 20	70	0.0632	0.603102	− 0.1743	0.2938
Min				− 0.2128	0.076951	− 0.4264	0.0233
Med				− 0.184	0.127337	− 0.4016	0.0533
Max				− 0.1106	0.361884	− 0.3369	0.1277
Range	Minimum	> 20	135	− 0.1546	0.073477	− 0.3153	0.0148
Min				0.1856	**0.03118**	0.0171	0.3437
Med				0.099	0.253053	− 0.0711	0.2636
Max				0.0396	0.648249	− 0.1302	0.2072
Range	Average	< 20	45	0.0096	0.950332	− 0.2848	0.3022
Min				− 0.1641	0.281351	− 0.4366	0.136
Med				− 0.1722	0.244305	− 0.4474	0.1227
Max				− 0.131	0.391008	− 0.4088	0.169
Range	Average	> 20	160	− 0.1392	0.079236	− 0.2881	0.0163
Min				0.2329	**0.003035**	0.0807	0.3746
Med				0.1327	0.094369	− 0.0229	0.2821
Max				0.0753	0.343693	− 0.0808	0.2278

*IOP* intraocular pressure [mmHg], *ICAS* iridocorneal angle size [°], *CI* confidence interval, *IOP range* difference between the lowest and highest IOP measured within 48 h, *min./med./max. IOP* minimum, median and maximum IOP measured within 48 h; bold numbers are statistically significant (*P* < 0.05)

### Subgroup analysis

Out of all measured 205 eyes, 44 eyes (21%) were diagnosed with glaucoma in the course of our examination. The remaining 161 eyes (79%) were used as “healthy” control group (HCG). Data (mean, standard deviation and range) of the evaluated parameters for the glaucoma- and the healthy control-subgroup are listed in Table 1: between these two subgroups only glaucoma relevant parameters (min, average, max and range of IOP; MD 30, slv 30, rim volume and corneal thickness) showed a statistically significant difference.

**Fig. 10:**
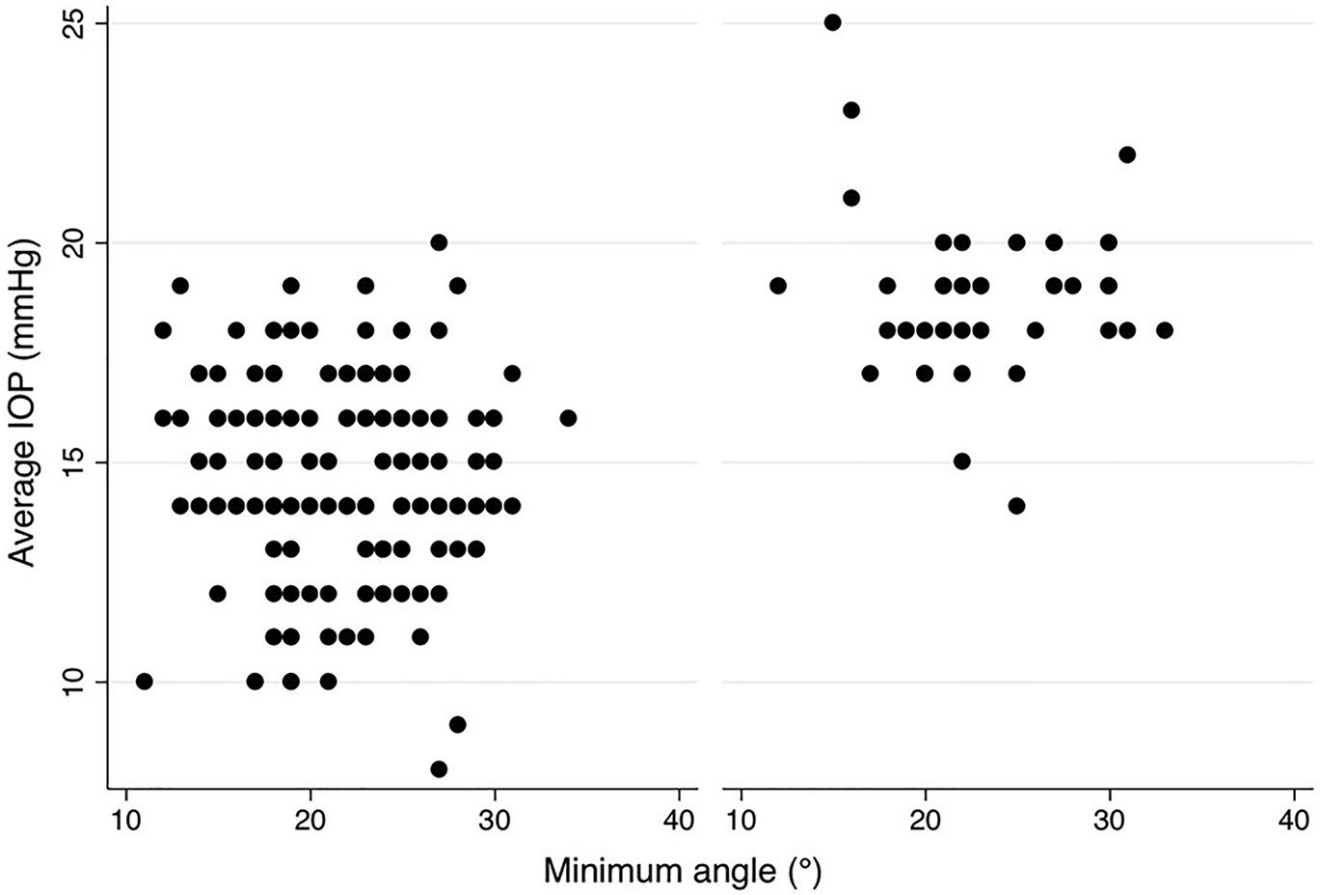
Distribution of average IOP and minimum iridocorneal angle in a HCG (on the left) and eyes with glaucoma (on the right). No significant impact of the iridocorneal angle on IOP

In the glaucoma cohort, mean IOP was 18.77 ± 1.86 mmHg and mean iridocorneal angle size 25.02° ± 4.96°. In the HCG cohort, mean IOP was 14.77 ± 2.25 mmHg and mean iridocorneal angle size 23.62° ± 4.64°. Subgroup analysis within the HCG (*P* = 0.45, *P* = 0.32, *P* = 0.84) and glaucoma cohort (*P* = 0.14, *P* = 0.75, *P* = 0.69) also showed no significant impact (Fig. 2). Figure 10 is showing that in the glaucoma group 23% of eyes had a minimum angle between 10° and 20°, and in the HCG even about 38% of eyes.

When we looked further on the effect of an ICAS between 10° and 20° or wider than 20° on the IOP range or maximum IOP in the glaucoma or healthy eyes, the results were as follows:

Within the glaucoma or healthy group itself, we could not find any effect of ICAS on maximum or range of IOP. This was found for both, minimum and average ICAS. Here, for an average angle of 10° to 20°, is restrictively to mention that we had only 6 eyes in the glaucoma group. Thus here statistical power is limited.

**Fig. 11:**
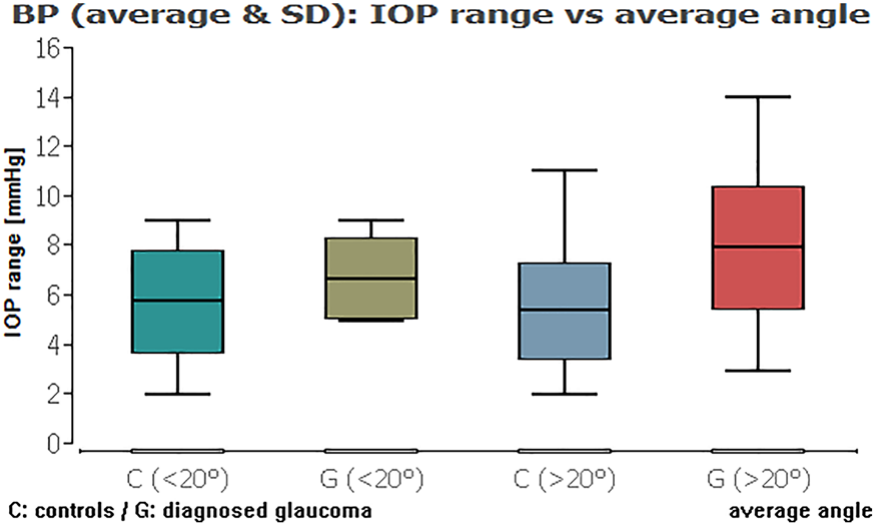
Boxplot of the IOP range in control and glaucoma group for an average ICAS narrower or wider than 20°. No significant impact of ICAS on IOP within the groups

**Fig. 12:**
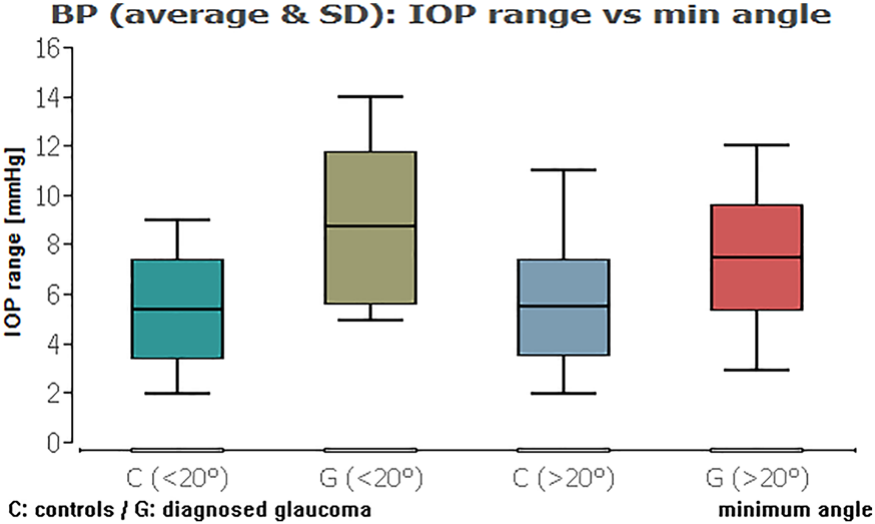
Boxplot of the IOP range in control and glaucoma group for the minimum ICAS narrower or wider than 20°. No significant impact of ICAS on IOP within the groups

**Fig. 13:**
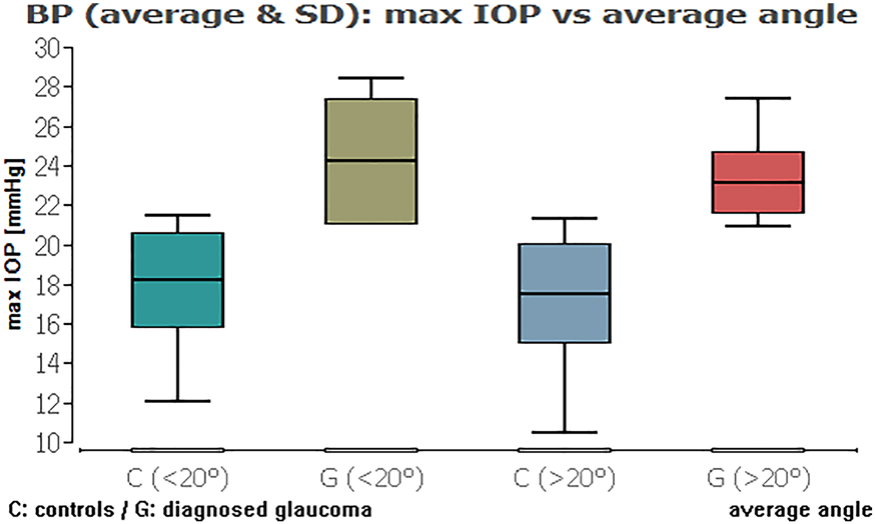
Boxplot of the maximum IOP in control and glaucoma group for an average ICAS narrower or wider than 20°. No significant impact of ICAS on IOP within the groups

**Fig. 14:**
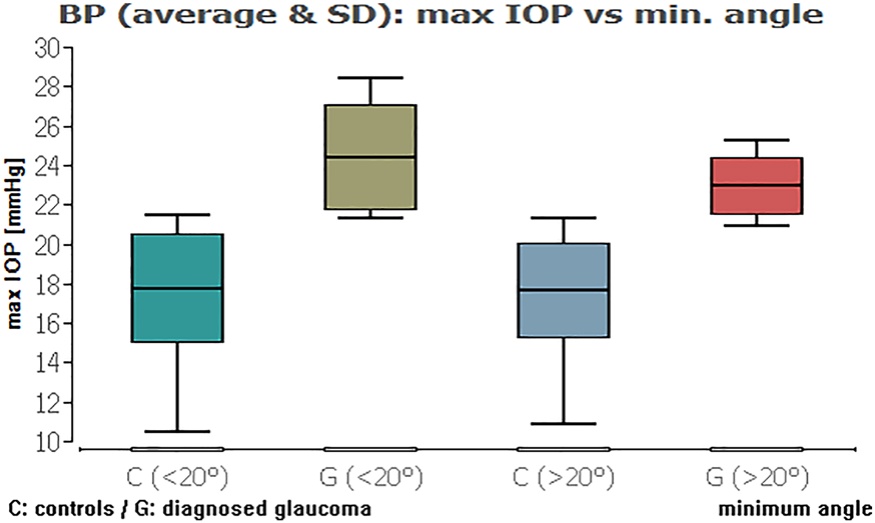
Boxplot of the maximum IOP in control and glaucoma group for the minimum ICAS narrower or wider than 20°. No significant impact of ICAS on IOP within the groups

When we compared the glaucoma and healthy groups against each other, except from the IOP range combined with average ICAS, there was always a highly statistically significant difference of the IOP data (Table 3; Figs. 11, 12, 13, 14 (box plots)). To sum up, there was in fact no significant effect of an ICAS in the range of 10° to 20° versus larger than 20° on the maximum IOP or IOP range within the groups but, as expected, a significant IOP difference between the glaucoma and healthy subgroup.

**Table 3:** Comparison of IOP range and maximum IOP in the healthy cohort (HCG) and eyes with glaucoma (G) in correlation to ICAS smaller or wider than 20°

	Group [HCG = healthy; G = glaucoma]	ICAS [°]	*n*	Minimum angle *P* = *	Average angle *P* = *
IOP range	HCG	< 20°	60	**0.0012**	0.3504
	G	< 20°	10		
Maximum IOP	HCG	< 20°	60	** < 0.0001**	** < 0.0001**
	G	< 20°	10		
IOP range	HCG	> 20°	101	** < 0.0001**	** < 0.0001**
	G	> 20°	34		
Maximum IOP	HCG	> 20°	101	** < 0.0001**	** < 0.0001**
	G	> 20°	34		
IOP range	HCG	< 20°	60	0.9086	0.2347
	HCG	> 20°	101		
Maximum IOP	HCG	< 20°	60	0.5464	0.1333
	HCG	> 20°	101		
IOP range	G	< 20°	10	0.3395	0.2180
	G	> 20°	34		
Maximum IOP	G	< 20°	10	0.1020	0.6048
	G	> 20°	34		

*ICAS* iridocorneal angle size [°], *IOP* intraocular pressure [mmHg] within 48 h

*Statistical mean comparison (Welch *t* test or Wilcoxon–Mann–Whitney test depending on data distribution); bold numbers are statistically significant (*P* < 0.05)

Comparing the HCG to the glaucoma group, there was a significant difference only in terms of IOP (*P* < 0.0001) but not for minimum ICAS (*P* = 0.27). Similarly, the anterior chamber volume and anterior chamber depth showed no significant impact on average (*P* = 0.27, *P* = 0.34) or maximum IOP (*P* = 0.48, *P* = 0.32). The difference between the glaucoma and HCG cohort was only significant in terms of average IOP (*P* < 0.001) but not for minimum ICAS (*P* = 0.07). Chi-square test showed no increased prevalence of IOP peaks of  > 21 mmHg within 48 h in eyes with an ICAS between 10° and 20° (P = 0.18).

## Discussion

In order to differentiate between glaucoma (suspect) eyes (SG) and healthy eyes with “variations of the norm”, we performed an in-patient measurement of diurnal tension curves (DTC). We were wondering, if the iridocorneal angle size (ICAS) of eyes would have an effect on the DTC. Thus, we investigated the effect of the ICAS data, measured by Scheimpflug technique, on the DTC.

As mentioned in the introduction, it is difficult to differentiate eyes with an early stage of glaucoma, where treatment would be recommended, to healthy eyes with sometimes higher IOP, where controls are adequate. Beside data of papilla excavation, visual field examination and RFNL measurements by OCT, IOP is the main risk factor for glaucoma and still plays a major role in the decision of treatment. To detect potentially existing IOP peaks, a DTC is appropriate. Here, we were interested, if the ICAS, measured by Scheimpflug technology, would show an effect on the obtained IOP data.

It is well reasoned that a smaller anterior chamber angle might have an impact on the IOP. If so, this might allow clinicians to predict e.g. IOP in patients and reduce the hospital stay. Our hypothesis, that a smaller angle might have an inverse effect on IOP or lead to higher frequency of IOP peaks within a 48-h pressure profile, was based on the fact that after cataract removal IOP decreases. So far it was assumed that this decrease is due to an increased iridocorneal angle.

Accurate topographic evaluation of the ICA, which is the angle between the iris and the cornea in the anterior chamber, is helpful in determining the risk for glaucoma [22–24]. The standard technique to evaluate the anatomical width of the ICA is still gonioscopy. Here, the Shaffer grading scheme is often used [9, 25], where a closed angle is grade 0, an angle ≤ 10° is grade 1, 10°–20° is grade 2, 20°–35° is grade 3, and larger is grade 4. An iridocorneal angle of less than 20° with only the Schwalbe line and the trabecular meshwork visible in the gonioscopy is defined as narrow [22], corresponding to a Shaffer angle grade 2.

Newer techniques of measuring the ACA are ultrasound biomicrosopy (UBM), Scheimpflug imaging (e.g. Pentacam) an anterior segment OCT (AS-OCT) with the advantage of a non-contact measurement in Pentacam and AS-OCT [26]. Although ACA data of Scheimpflug imaging and AS-OCT are comparable, measured data of narrow angles with Scheimpflug devices are limited due to issues in visualising the most peripheral part of the iris [27, 28].

Recent publications dealing with OCT measurements of the anterior chamber angle ranges between 31.8° ± 7.49° (for hyperopic eyes) to 40.8° ± 8.1° (for myopic eyes) [29] and 35.9° ± 5.7° [30]. These data are in line with our manually measured ACA (here for all eyes of in the mean 23.9° ± 4.7°), because Shajari et al. had found an average difference of 11.4° to 12.1° that has to be added from manually to automatically measured angles [21].

Mansberger et al. [31] reported that “cataract surgery decreases IOP in patients with ocular hypertension over a long period of time”, even when all eyes had an open iridocorneal angles before the operation. On the other hand, it is easy to comprehend, that eyes with a narrow anterior chamber angle could benefit more from the pressure lowering effect of a cataract operation, as pointed out by Shrivastava and Singh [32]. Compared to our research, there should be more pressure lowering effects after cataract operations than the increase in iridocorneal angle size.

In comparison with a study of Sanchez-Parra et al. [33], in which they reported an inverse relation between the diurnal IOP fluctuation and the anterior chamber angle, we could not confirm these results in our cohort. The findings of Baskaran et al. [34], who reported higher IOP variability in patients with primary angle-closure glaucoma (PACG) compared to angle-closure suspects (PACS) and normal controls, are understandable because of the even smaller ICAS in PACG subjects.

The analysis of our data showed that an angle size of larger than 10° had no impact on the IOP of the entire group as well as in the subgroup analysis of the healthy cohort, and patients diagnosed with glaucoma. It is noteworthy that patients with ICAS between 10° and 20°, which is relatively narrow, but still counted as open-angle subjects, showed no difference in the eye pressure levels within the overall cohort, the healthy or the glaucoma patients group in comparison to eyes with an ICAS larger than 20°. We also could not find any difference in the results performing the analysis on just the right eye throughout all patients, therefore finding no inter-collinearity.

Little is known about the exact iridocorneal angle size (ICAS) in PACG as most of the published literature on PACG covers other parameters such as anterior chamber depth (ACD), angle opening distance (AOD) or lens thickness (LT). Aksoy NÖ et al. [35] reported a mean ACA of 24.2° ± 2.6° in the PACG group vs. 30.5 ± 2.3 in the control group measured by dual Scheimpflug imaging. Leong et al. [36] published a trabecular-iris angle (TIA) of 6.8° ± 4.9° before and of 14° ± 6.7° after laser peripheral iridoplasty (LPI) measured by AS-OCT what is in line with post-LPI data of Ma et al. [37] for anterior chamber angle (ACA) values from 12.91° ± 6.31° to 16.45° ± 5.87° (depending on the measuring position) after a laser peripheral iridotomy.

It would be interesting to clarify how small the ICAS have to be to “really” increase the IOP. In accordance to our findings (see scattergram in Fig. 2) and the above mentioned ACA data of Leong et al., where an ACA of < 10° before LPI was described, an ACA smaller 10°, equivalent to a Shaffer Grade 1, seems to be a critical value. Additional effects on the efflux resistance of the aqueous humour, like peripheral anterior synechiae or minimal anatomical changes of the ICA, should be considered in later examinations.

ICAS smaller than 10 degree are, due to technical limitations, not correctly measurable by a Scheimpflug device. This might be a limitation of our study if the above-mentioned assumption is true because the critical small ICAS data would not be detectable. Therefore, further investigations in PACG eyes with multiple measurements by AS-OCT systems, having a technical advantage in measuring very small ACAs, could be useful to establish a lower limit of ICAS, where IOP peaks are more likely. If such a critical limit of ICAS would be established, this might help to indicate, when a prophylactic LPI in PACS eyes is reasonable. This could avoid IOP peaks and thus minimize the progression of glaucomatous damages.

Further it is a limitation of the study that the Pentacam measurements were only taken once. Thus, it is not possible to evaluate the effect of different pupil sizes on ICAS and therefore on IOP changes. Pentacam and IOP data were only measured in sitting position of the patients. Thus, our findings are possible not transferrable to other body positions. An additional limitation might be that the manual measurements of ICAS were not generally cross-checked by another person to examine the interindividual collinearity and that the GAT were done by different ophthalmologists.

Furthermore, the absence of a standard control is a limitation, but insurance restrictions prevented us from performing a 48-h pressure profile on an in-patient situation, as we did in our cohort. Nevertheless, we believe that our conclusions are noteworthy because there were no dependency for ICAS larger than 10° and IOP.

## Conclusion

Diurnal tension curves seem to be independent of ICAS, at least for angles larger than 10° in open-angle subjects. Although diurnal IOP pressure profiles play an important role in the diagnosis of glaucoma, angle parameter measurements cannot be used to predict IOP diurnal fluctuations.

## Data Availability

The datasets generated during the current study are available from the corresponding author on reasonable request.

## References

[1] Kahn HA, Milton RC (1980) Alternative definitions of open-angle glaucoma. Effect on prevalence and associations in the Framingham eye study. Arch Ophthalmol 98(12):2172–2177. 10.1001/archopht.1980.01020041024003. 10.1001/archopht.1980.01020041024003 7447769

[2] Fechtner RD, Weinreb RN (1994). Mechanisms of optic nerve damage in primary open angle glaucoma. Surv Ophthalmol.

[3] Cockburn DM (1985). Glaucoma enigma. Am J Optom Physiol Opt.

[4] Gottlieb LK, Schwartz B, Pauker Sg (1983) Glaucoma screening. A cost-effectiveness analysis. Surv Opthalmol 28(3):206–226. 10.1016/0039-6257(83)90098-x. 10.1016/0039-6257(83)90098-x 6422576

[5] Kaushik S, Pandav S, Ram J (2003). Neuroprotection in glaucoma. J Postgrad Med.

[6] Weinreb RN, Khaw PT (2004). Primary open-angle glaucoma. Lancet.

[7] Weinreb RN, Aung T, Medeiros FA (2014). The pathophysiology and treatment of glaucoma. JAMA.

[8] Wolfs RC, Borger PH, Ramrattan RS, Klaver CC, Hulsman CA, Hofman A, Vingerling JR, Hitchings RA, de Jong PT (2000) Changing views on open-angle glaucoma: definitions and prevalences: the Rotterdam Study. Invest Ophthalmol Vis Sci 41(11):3309–3321 11006219

[9] Shaffer RN (1960). Primary glaucomas. Gonioscopy, ophthalmoscopy and perimetry. Trans Am Acad Ophthalmol Otolaryngol.

[10] Jonas JB, Budde WM, Stroux A (2012). Diurnal intraocular pressure profiles in chronic open-angle glaucoma. Asia Pac J Ophthalmol (Phila).

[11] Jonas JB, Budde W, Stroux A (2005). Single intraocular pressure measurements and diurnal intraocular pressure profiles. Am J Ophthalmol.

[12] Kitazawa Y, Horie T (1975). Diurnal variation of intraocular pressure in primary open-angle glaucoma. Am J Ophthalmol.

[13] David R, Zangwill L, Briscoe D (1992). Diurnal intraocular pressure variations: an analysis of 690 diurnal curves. Br J Ophthalmol.

[14] Liu JHK, Bouligny RP, Kripke DF (2003). Nocturnal elevation of intraocular pressure is detectable in the sitting position. Invest Ophthalmol Vis Sci.

[15] Liu JH, Kripke DF, Hoffman RE (1998). Nocturnal elevation of intraocular pressure in young adults. Invest Ophthalmol Vis Sci.

[16] Wilensky JT (1991). Diurnal variations in intraocular pressure. Trans Am Ophthalmol Soc.

[17] Qureshi IA, Xiao RX, Yang BH (1999). Seasonal and diurnal variations of ocular pressure in ocular hypertensive subjects in Pakistan. Singapore Med J.

[18] Kohlhaas M, Boehm AG, Spoerl E, Pürsten A, Grein JH, Pillunat LE (2006). Effect of central cormeal thickness, corneal curvature, and axial length on applanation tonometry. Arch Ophthalmol.

[19] Kohlhaas M, Spoerl E, Boehm AG, Pollack K (2006) A correction formula for the real intraocular pressure after LASIK for the correction of myotic astigmatism. J Refract Surg 22(3):263–267. 10.3928/1081-597X-20060301-11 10.3928/1081-597X-20060301-11 16602315

[20] European Glaucoma Society (2020) Terminology and guidelines for glaucoma, 5th edn. www.eugs.org/eng/guideline.asp 10.1136/bjophthalmol-2021-egsguidelines 34675001

[21] Shajari M, Herrmann K, Bühren J, Vuunava P, Vounotrypidis E, Müller M, Al Khateeb G, Kohnen T (2019). Anterior chamber angle, volume and depth in a normative cohort—a retrospective cross-sectional study. Curr Eye Res.

[22] Lowe RF (1970). Aetiology of the anatomical basis for primary angle-closure glaucoma. Biometrical comparisons between normal eyes and eyes with primary angle-closure glaucoma. Br J Ophthalmol.

[23] Sihota R, Lakshmaiah NC, Agarwal HC (2000). Ocular parameters in the subgroups of angle closure glaucoma. Clin Exp Ophthalmol.

[24] George R, Paul PG, Baskaran M (2003). Ocular biometry in occludable angles and angle closure glaucoma: a population based survey. Br J Ophthalmol.

[25] Shaffer RN (1973). A suggested anatomic classification to define pupillary block glaucomas. Invest Ophthalmol.

[26] Friedman DS, He M (2008). Anterior chamber angle assessment techniques. Surv Ophthalmol.

[27] Kurita N, Mayama C, Tomidokoro A, Aihara M, Araie M (2009). Potential of the pentacam in screening for primary angle closure and primary angle closure suspect. J Glaucoma.

[28] Grewal DS, Brar GS, Jain R, Grewal SPS (2011) Comparison of Scheimpflug imaging and spectral domain anterior segment optical coherence tomography for detection of narrow angle chamber angles. Eye 25:603–611 10.1038/eye.2011.14 PMC3171268 21336254

[29] Vossmerbäumer U, Schuster AK, Fischer JE (2013). Width of anterior chamber angle determined by OCT, and correlation to refraction and age in a German working population: the MIPH Eye & Health Study. Graefes Arch Clin Exp Ophthalmol.

[30] Müller M, Dahmen G, Pörksen E, Geerling G, Laqua H, Ziegler A, Hoerauf H (2006). Anterior chamber angle measurement with optical coherence tomography: intraobserver and interobserver variability. J cataract Refract Surg.

[31] Mansberger SL, Gordon MO, Jampel H (2012). Reduction in intraocular pressure after cataract extraction: the Ocular Hypertension Study Group. Ophthalmology.

[32] Shrivastava A, Singh K (2010). The effect of cataract extraction on intraocular pressure. Curr Opin Ophthalmol.

[33] Sanchez-Parra L, Pardhan S, Buckley RJ (2015). Diurnal intraocular pressure and the relationship with swept-source OCT–derived anterior chamber dimensions in angle closure: the IMPACT StudyDiurnal IOP in PAC and PACS: the IMPACT study. Invest Ophthalmol Vis Sci.

[34] Baskaran M, Kumar RS, Govindasamy CV (2009). Diurnal intraocular pressure fluctuation and associated risk factors in eyes with angle closure. Ophthalmology.

[35] Aksoy NÖ, Cakir B, Dogan E, Alagoz G (2018). Evaluation of anterior segment parameters in pseudoexfoliative glaucoma, primary angle-closure glaucoma, and healthy eyes. Turk J Ophthalmol.

[36] Leong JCY, O'Connor J, Ang GS, Wells AP (2014). Anterior segment optical coherence tomography changes to the anterior chamber angle in the short-term following laser Peripheral Iridoplasty. J Curr Glaucoma Pract.

[37] Ma X-Y, Zhu D, Zou J (2016). Comparison of ultrasound biomicroscopy and spectral domain anterior segment optical coherence tomography in evaluation of anterior segment after laser peripheral iridotomy. Int J Ophthamlol.

